# A rapid and simple clonality assay for bovine leukemia virus-infected cells by amplified fragment length polymorphism (AFLP) analysis

**DOI:** 10.1128/spectrum.01714-24

**Published:** 2024-11-21

**Authors:** Tomoko Kobayashi, Sakurako Makimoto, Nagaki Ohnuki, Md Belal Hossain, M. Ishrat Jahan, Misaki Matsuo, Kazuhiko Imakawa, Yorifumi Satou

**Affiliations:** 1 Department of Animal Science, Faculty of Agriculture, Tokyo University of Agriculture, Kanagawa, Japan; 2 Division of Genomics and Transcriptomics, Joint Research Center for Human Retrovirus Infection, Kumamoto University, Kumamoto, Japan; 3 Department of Food Microbiology, Faculty of Nutrition and Food Science, Patuakhali Science and Technology University, Dumki, Patuakhali, Bangladesh; 4 Laboratory of Molecular Reproduction, Research Institute of Agriculture, Tokai University, Kumamoto, Japan; University of Florida College of Dentistry, Gainesville, Florida, USA

**Keywords:** Bovine leukemia virus, clonality, enzootic bovine leukosis, retrovirus, AFLP

## Abstract

Enzootic bovine leukosis (EBL), although eradicated in some European countries, is still the most common neoplastic disease of cattle, caused by the bovine leukemia virus (BLV). During the progression of EBL, BLV-infected cells clonally expand, and some of which result in tumor onset. The clonality of BLV-infected cells is generally evaluated with NGS or Sanger sequencing. Although these methods clearly distinguish EBL from non-EBL cases, the procedures are complex and not practical for routine veterinary diagnosis. In this study, we developed an amplified fragment length polymorphism (AFLP) analysis for BLV clonality assay (BLV-AFLP). This analysis uses restriction enzyme digestion to amplify the chimeric regions of BLV 3’ linear transcribed region (LTR) and host genome through conventional polymerase chain reaction (PCR) and visualizes the results by gel-electrophoresis. The method was established using cattle samples representing different stages of the disease: BLV-uninfected, non-EBL, and EBL cattle. Non-EBL cattle showed smeared bands, indicating polyclonal proliferation, while EBL cattle showed distinct bands, indicating clonal expansion. The results of BLV-AFLP correlated well with those of previously reported methods, suggesting its efficacy in detecting clonal proliferation. The validation using blood samples of non-EBL cattle and tumor samples of EBL cattle confirmed that BLV-AFLP could effectively identify clonal proliferation in EBL samples. Moreover, the emergence of dominant clones in the tumor at later stages was successfully detected before EBL onset in some cattle, highlighting its sensitivity and potential for early detection. Overall, BLV-AFLP is suitable for practical use in the field, improving BLV management strategies and minimizing economic losses.

**IMPORTANCE:**

Enzootic bovine leukosis (EBL) is routinely diagnosed based on external manifestations at the farm, such as the presence of tumors and/or general lymph node enlargement. However, due to the nonspecific clinical manifestations of EBL, over half of EBL cases are unrecognized at the farm, with most cases being diagnosed during postmortem inspection at the slaughterhouse. Early detection and monitoring of clonal expansion are necessary for managing EBL and reducing economic losses. In this study, we developed BLV-AFLP that represents a significant advancement in the diagnosis of EBL in cattle. This method can rapidly assess the clonal proliferation of BLV-infected cells, crucial for distinguishing between asymptomatic and EBL cattle. Additionally, tracking clonal dynamics offers insights into the disease’s progression, potentially providing strategies for avoiding economic losses. Overall, as BLV-AFLP is a simple and rapid test for detecting EBL, it is feasible and efficient for routine veterinary practice.

## INTRODUCTION

Enzootic bovine leukosis (EBL), although eradicated in some European countries, is still the most common neoplastic disease of cattle caused by the bovine leukemia virus (BLV) (1–3). BLV belongs to Deltaretrovirus of the family Retroviridae. Similar to other retroviruses, following BLV infection, the RNA genome is reverse-transcribed into DNA and integrated into the host genome. This process, known as the “infectious cycle,” results in BLV integration sites being distributed at various locations in the host genome, producing thousands of BLV-infected cell clones with unique integration site(s). Previous studies reported that during primary infection, various integration sites are detected in BLV-infected cattle (4, 5). In addition to the infectious cycle, there is another infection process termed as the “mitotic cycle,” which relies on cell proliferation. Thus, the majority of the infected clones were produced during the period of primary infection by the infectious cycle. After a long incubation period, a few of these clones expand through the mitotic cycle, leading to lymphosarcoma onset (4). In the EBL stage, the peripheral blood and/or tumor tissues consist of one to four predominant clone(s) (6, 7).

Most BLV-infected cattle remain clinically asymptomatic throughout their lifetime, while about 30% of BLV-infected cattle develop persistent lymphocytosis (PL), which is characterized by nonmalignant polyclonal expansion of BLV-infected B cells. Less than 5% of BLV-infected cattle progress to EBL. The clinical signs of EBL vary depending on where tumors are located, including digestive disturbances, inappetence, weight loss, weakness, and emaciation (8). Since these clinical signs are not specific to EBL, over half of EBL cases remain unrecognized at the farm and most EBL cases are diagnosed at postmortem inspection at the slaughterhouse by the recognition of evident lymphoma in internal tissues or lymph nodes. Clonality assays have improved clinical diagnosis by detecting the presence of predominant clones in EBL cattle. In our previous study, we evaluated the clonality of BLV-infected cells using a next-generation sequencing (NGS)-based method (BLV-capture-seq), which uses specific capture probes of the BLV genome (7). This approach effectively differentiates EBL cattle from non-EBL cattle by analyzing the diversity of the host sequences adjacent to proviral sequences. In non-EBL cattle, diverse host–virus chimera sequences were detected, indicating polyclonal expansion, while one to four predominant clone(s) were detected in EBL cattle, indicating clonal expansion of infected cells. However, this approach contains complex reaction steps and requires specialized facilities and laboratory skills. We further integrated a novel approach, Rapid Amplification of Integration Site (RAIS), and developed BLV-RAIS for the quick analysis of BLV clonality (6, 9). This method allows PCR amplification of the chimeric region of BLV and the host genome. EBL cattle were clearly distinguished from non-EBL cattle by clonality values calculated with sequencing data, and the results of BLV-RAIS analysis were highly correlated to those of NGS-based BLV-capture-seq.

While BLV-RAIS is a simple and rapid method compared to BLV-capture-seq, it is still impractical for routine veterinary clinical testing due to the requirement for multiple reaction steps, sequencing, and software analysis of sequencing data. In this study, we modified the method in order to analyze the clonality of clinical samples by conventional PCR and agarose-gel electrophoresis. To obtain the fragment of the chimeric region of BLV 3’LTR and the host genome, we employed amplified fragment length polymorphism analysis for BLV clonality assay (BLV-AFLP). The results of BLV-AFLP analysis demonstrated data comparable to those obtained by BLV-RAIS or BLV-capture-seq.

## RESULTS

### Establishment of BLV-AFLP analysis for detecting the clonality of BLV-infected cells

**Fig 1:**
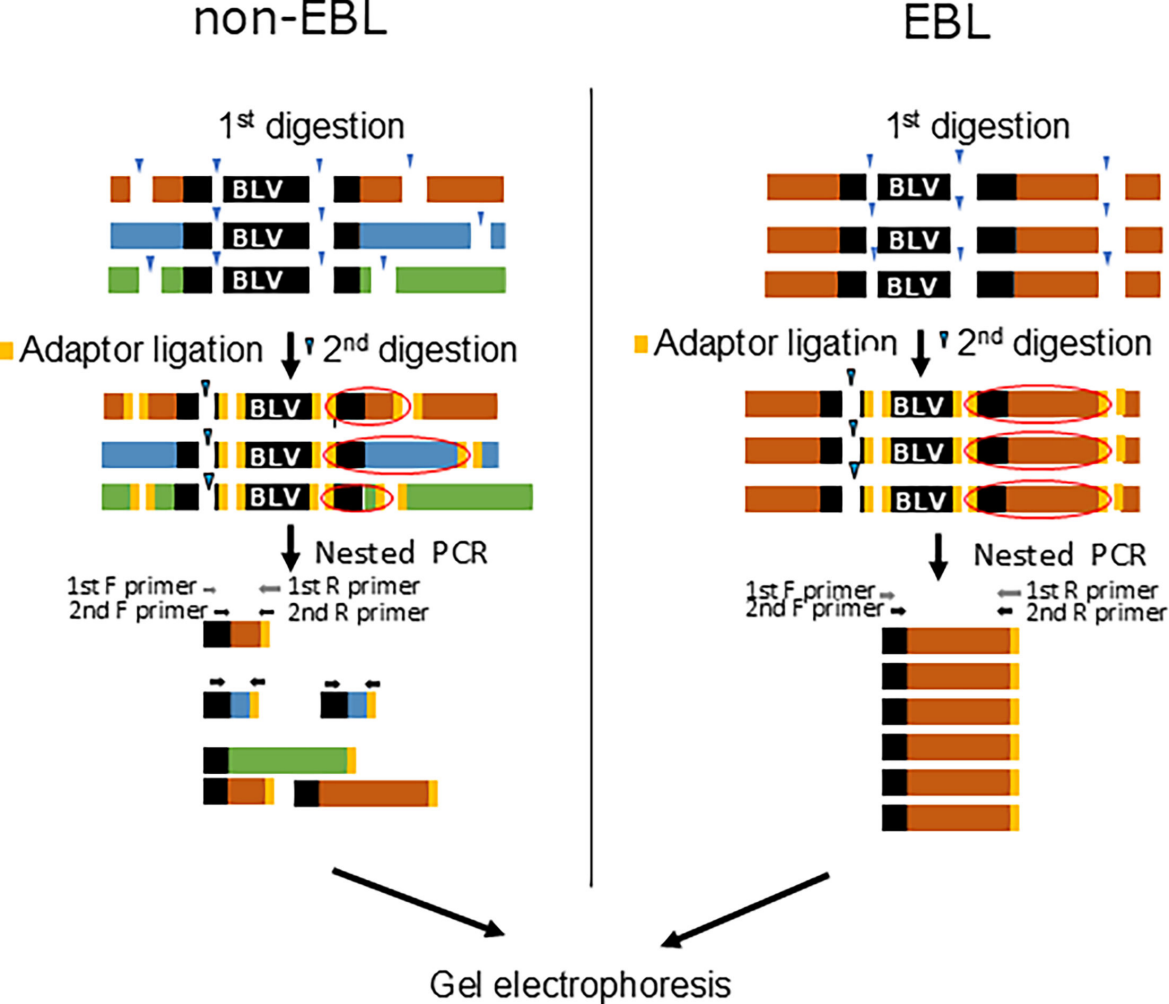
Schematic diagram of BLV-AFLP analysis detecting the clonality of BLV-infected cells. The restriction enzyme digestion and adapter ligation were conducted for preparation of DNA libraries. Following second restriction enzyme digestion, the first and second PCR were performed using forward primers that bind to the 3’LTR of the provirus (black) and reverse primers that bind to the adapter sequence (yellow). The bovine genome sequence flanking BLV3’LTR is unique to each infected cell lineage. Therefore, the length of the restriction fragments including BLV 3’LTR and the host genome varies according to the restriction site of the bovine genome sequence. The size and the number of PCR products reflect each infected cell clone lineage, which enables visualization of clone diversity by gel electrophoresis. Orange, blue, and green bars represent host sequences that are derived from individual infected-clones. The blue arrow represents restriction enzyme sites.

To visually detect the clonality of BLV-infected cells by agarose gel electrophoresis, restriction enzymes were used for preparation of DNA libraries (Fig. 1). According to the previous report, the four nucleotide recognition enzyme, *Rsp*RS II, was selected (10, 11). In the BLV 3’LTR sequence, the recognition site is located at the conserved region of all BLV genotypes, with no restriction enzyme site downstream of the primer annealing region. Next, the adapters were ligated to restriction fragments using a DNA library preparation kit. In the BLV genome, there is an *Rsp*RS II restriction enzyme site in 156 bp downstream of 5’LTR, which generates fragments that can be unintentionally amplified by nested-PCR. To avoid this amplification, the DNA library was further digested with *Fba* I after the adapter ligation step. In order to reduce the overall reaction time, *Mse* I, an isoschizomer of *Rsp*RS II, and *Bcl* I, an isoschizomer of *Fba* I, which are capable of more rapid digestion, were also used for the analysis. The fragments containing the BLV 3’LTR and the host genome were specifically amplified by the forward primer targeting the 3’ LTR and the reverse primer targeting the adapter sequence ligated to the 3’ end of the fragments. The second PCR was performed using a nested forward primer that targeted an internal region to the first forward primer. The host genome sequence flanking BLV 3’LTR is unique to each infected cell lineage. Therefore, the length of the restriction fragments varies according to the restriction site of the bovine genome sequence. As a result, the size and the number of PCR products reflect each infected cell clone lineage, which enables visualization of clone diversity by gel electrophoresis.

### Analysis of clonal proliferation of BLV-infected cells by agarose gel electrophoresis

**Fig 2:**
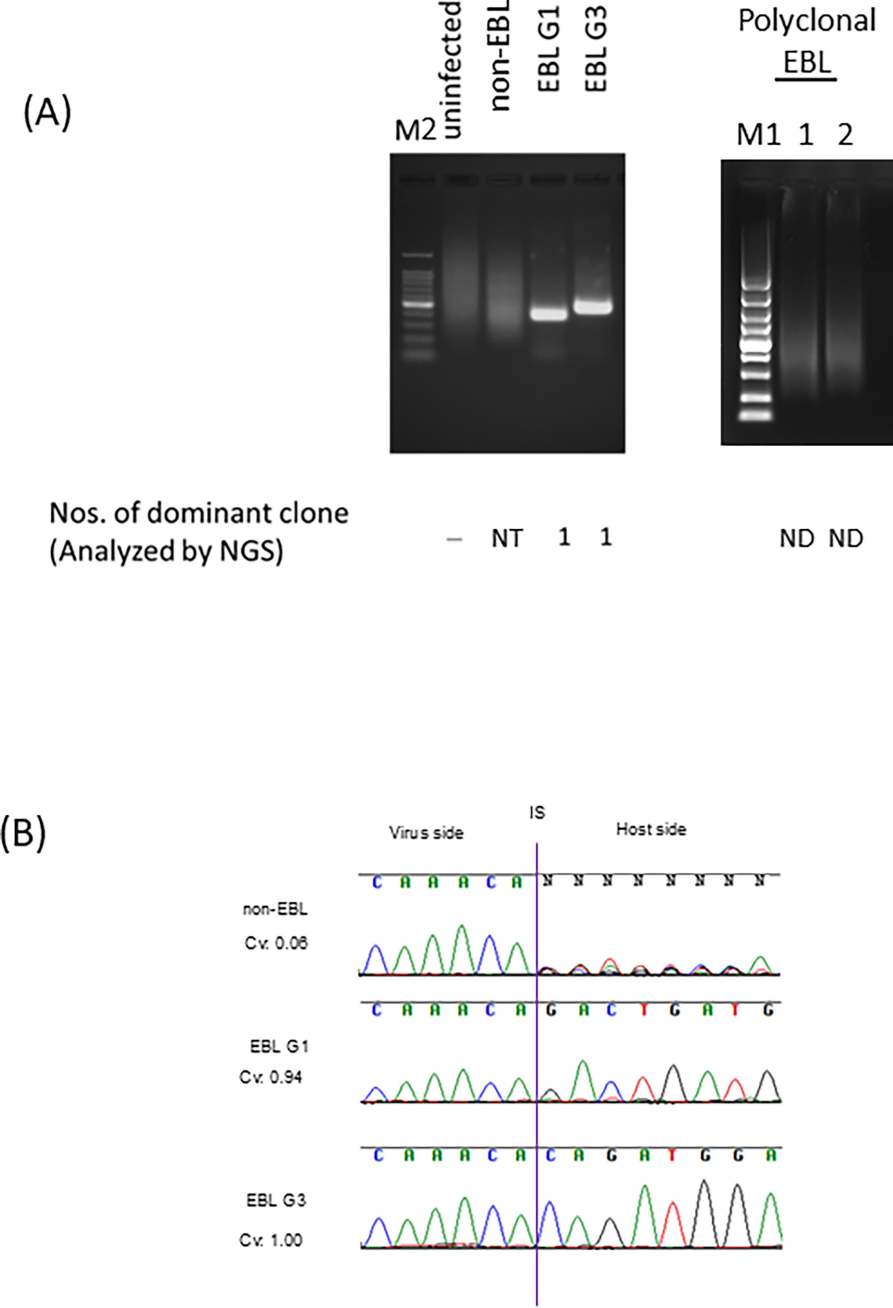
Analysis of the clonal proliferation of BLV-infected cells by agarose gel electrophoresis (**A**) Genomic DNA of six cattle samples representing different disease stages was analyzed by BLV-AFLP: BLV-uninfected, non-EBL, two EBL cattle with different genotypes (genotypes 1 and 3), and two EBL cattle with multiple clones. The numbers of dominant clones were detected by BLV-capture-seq. NT: not tested; ND: not detected (**B**) The base calling data of provirus 3’LTR end and host chimeric region analyzed by BLV-RAIS. The sequence of BLV 3’LTR is the same in all amplicons, while the sequence of the cattle sequence varies according to the restriction site. IS: integration site, Cv: clonality values calculated by BLV-RAIS. M1: 5,000-bp ladder marker, M2: 100-bp ladder marker.

To assess the clonal proliferation of BLV-infected cells by BLV-AFLP, the genomic DNA of four cattle samples representing different stages of the disease was analyzed: BLV-uninfected, non-EBL, two EBL cattle with clonal proliferation of different genotypes (genotypes 1 and 3), and two EBL cattle with polyclonal proliferation. These samples were fragmentated with restriction enzymes, and adapters were ligated. Subsequently, nested-PCR, as described above, was performed, and amplified PCR products were analyzed by agarose gel electrophoresis (Fig. 2A). Smeared bands were observed in samples derived from non-EBL cattle and polyclonal EBL cattle, while distinct single bands were observed in samples from two EBL cattle. These results indicate that restriction fragments containing BLV 3’LTR and the host genome of non-EBL cattle and polyclonal EBL cattle vary in size, suggesting polyclonal proliferation of BLV-infected cells. On the other hand, restriction fragments of EBL cattle contain a large number of specific sizes in both genotypes 1 or 3, suggesting clonal proliferation of single predominant clones. Previously, we analyzed these non-EBL cattle and EBL cattle samples by BLV-capture-seq and polyclonal proliferation in the non-EBL cattle and two EBL cattle, respectively, and clonal proliferation in the EBL cattle samples infected with genotypes 1 and 3 was detected (7). To further confirm these results, the BLV-RAIS method was applied to these samples. The base calling data of Sanger sequencing showed various host sequences of non-EBL cattle. In contrast, a single peak of base calling data was detected in EBL cattle samples (Fig. 2B). Clonality values (Cvs) were calculated using Clova software (12) from signal and noise values of these data, and the values were 0.06 for non-EBL cattle and 0.94 and 1.00 for EBL cattle samples infected with genotypes 1 and 3, respectively (Fig. 2B). These results indicate that BLV-AFLP clearly distinguished non-EBL cattle from EBL cattle with clonal proliferation by analyzing clonal proliferation using electrophoresis.

### Clonal analysis by BLV-AFLP of BLV-infected cattle in the field

**Fig 3:**
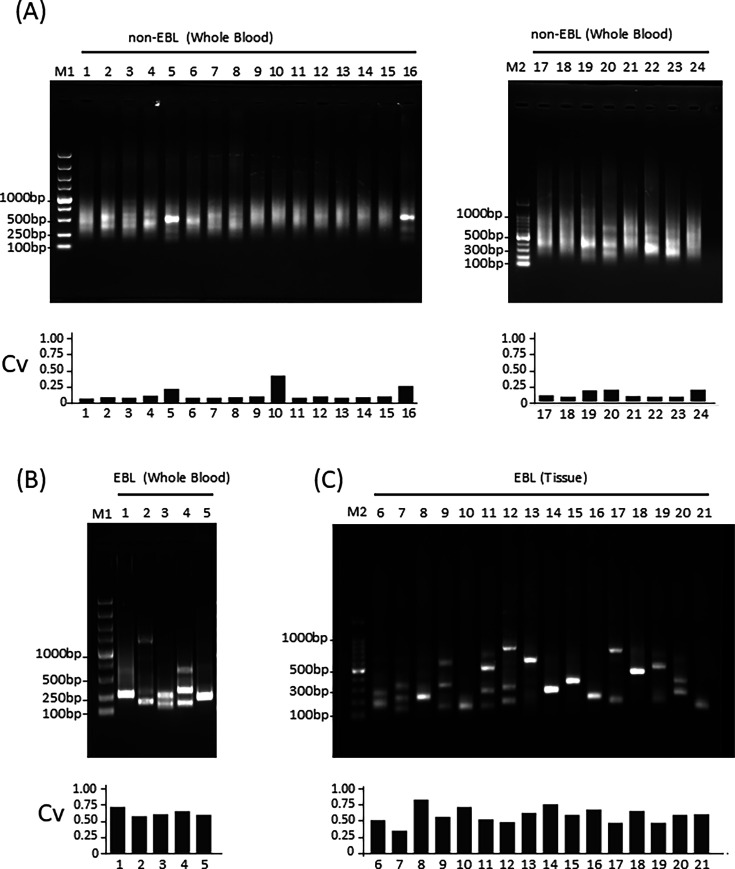
Validation of BLV-AFLP by analyzing BLV-infected cattle in the farm. The genomic DNA was extracted from blood that was drawn from 24 non-EBL cattle (A), five EBL cattle (B), and the tumor tissues of 16 EBL cattle (C). DNA samples were analyzed by BLV-AFLP. The black bar indicates the clonality value (Cv) of each sample calculated by BLV-RAIS. M1: 5,000-bp ladder marker, M2: 100-bp ladder marker.

To further validate this method, genomic DNA was extracted from blood of 24 non-EBL cattle, five EBL cattle, and from the tumor tissues of 16 EBL cattle (Table 1). These samples were analyzed by BLV-AFLP to visually detect the clonal expansion. The electrophoresis of PCR products from non-EBL cattle samples showed smeared bands, indicating polyclonal proliferation of BLV-infected cells (Fig. 3A). Conversely, while showing slight background smeared bands, the electrophoresis of PCR products from EBL blood and tumor samples revealed distinct bands, indicating clonal or oligoclonal proliferation of BLV-infected cells (Fig. 3B and C). Clonal expansion of these samples was calculated by BLV-RAIS. EBL cattle samples show Cvs above the cutoff value of 0.4, which were notably higher than those of non-EBL cattle. Some non-EBL cattle samples exhibited distinct background smeared bands (Fig. 3A, Nos 5, 16, 19, and 20), corresponding to relatively high Cvs, although those Cvs are below the cutoff value. Since the elevated Cvs of BLV-RAIS reflect the initiation of clonal expansion, these results suggest that the band patterns of BLV-AFLP represent the clonal proliferation of infected cells. However, in a few cases, however, high Cvs were observed with smeared band (non-EBL, No. 10).

**TABLE 1:** Detailed information of cattle analyzed in Fig. 3

	No.	Breed	Sample	PVL^*a*^
Non EBL	1	Holstein	Whole blood	143,908
	2	Holstein	Whole blood	119,906
	3	Holstein	Whole blood	89,924
	4	Holstein	Whole blood	143,908
	5	Holstein	Whole blood	51,353
	6	Holstein	Whole blood	81,925
	7	Holstein	Whole blood	67,936
	8	Holstein	Whole blood	33,091
	9	Holstein	Whole blood	65,124
	10	Holstein	Whole blood	36,978
	11	Holstein	Whole blood	58,135
	12	Holstein	Whole blood	11,048
	13	Holstein	Whole blood	23,733
	14	Holstein	Whole blood	19,777
	15	Holstein	Whole blood	16,486
	16	Holstein	Whole blood	18,739
	17	Holstein	Whole blood	100,248
	18	Holstein	Whole blood	54,115
	19	Holstein	Whole blood	69,405
	20	Holstein	Whole blood	80,152
	21	Holstein	Whole blood	86,391
	22	Holstein	Whole blood	28,902
	23	Holstein	Whole blood	64,404
	24	Holstein	Whole blood	106,308
EBL	1	Holstein	Whole blood	NA^*b*^
	2	Japanese Black	Whole blood	NA
	3	Holstein	Whole blood	NA
	4	Japanese Black	Whole blood	246,306
	5	Japanese Black	Whole blood	210,604
	6	Holstein	Heart	NA
	7	Holstein	Heart	NA
	8	Holstein	Heart	NA
	9	Crossbreed	Heart	NA
	10	Japanese Black	Heart	NA
	11	Japanese Black	Heart	NA
	12	Holstein	Heart	NA
	13	Holstein	Heart	NA
	14	Holstein	Uterus	NA
	15	Holstein	Uterus	NA
	16	Holstein	Heart	NA
	17	Holstein	Heart	NA
	18	Holstein	Heart	NA
	19	Holstein	Heart	NA
	20	Holstein	Lymph nodes	NA
	21	Japanese Black	Lymph nodes	NA

^*a*^ Proviral load (copies/10^5^cells)

^*b*^ Not available.

### Clone dynamics over the time course in EBL cattle

**Fig 4:**
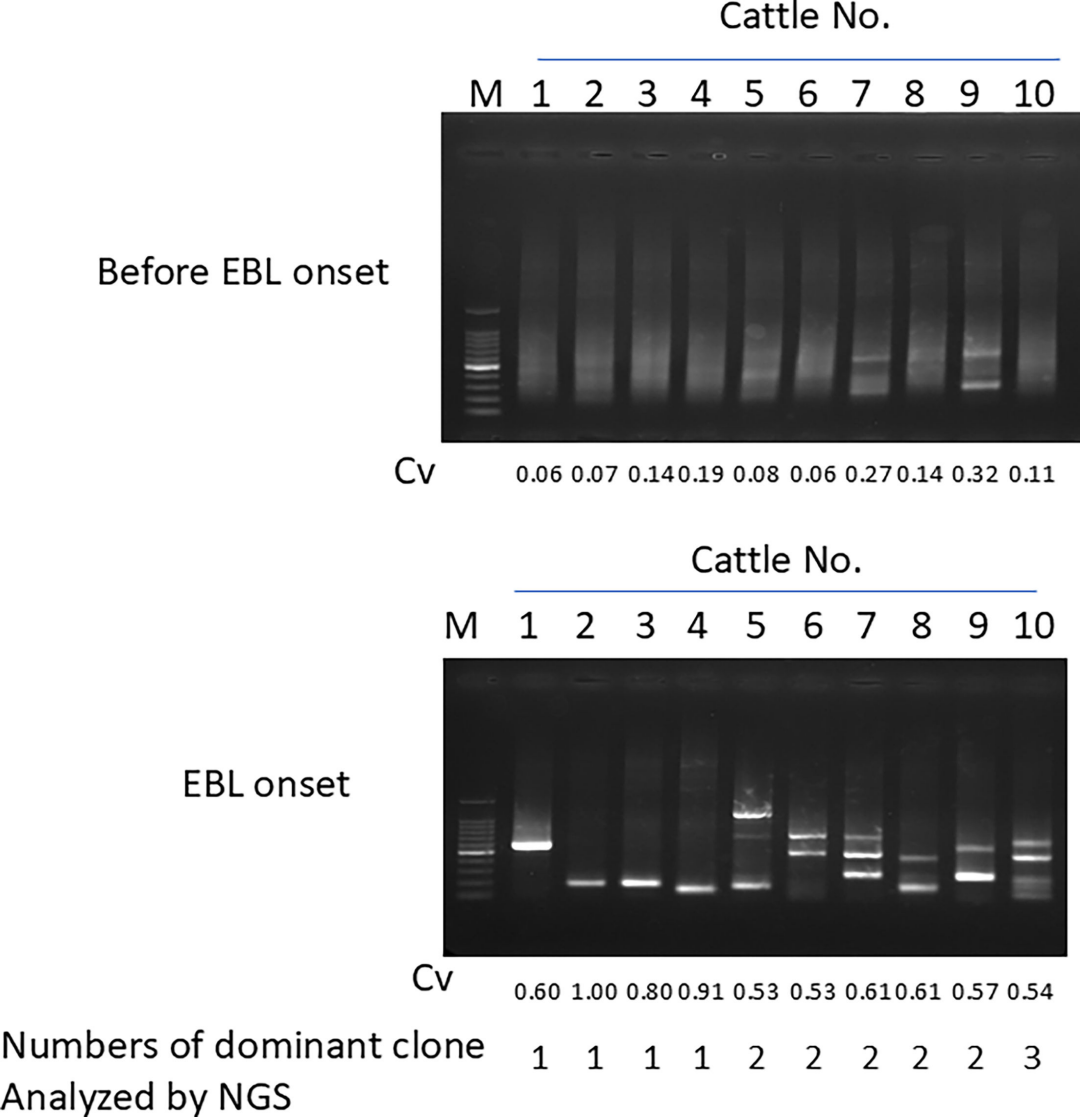
Clonal proliferation of BLV-infected cells during the oncogenic process in a longitudinal cohort study. The gel electrophoresis of BLV-AFLP of individual BLV-infected cells in PBMCs and tumor tissues (EBL onset) of 10 cattle is shown. The Cvs calculated by BLV-RAIS and the numbers of BLV integration sites analyzed by BLV-capture-seq are shown below.

To confirm whether this method can track the emergence of predominant clones in EBL cattle, we analyzed cohort samples of EBL cattle (Fig. 4). Tumor samples from EBL cattle were collected at the slaughterhouse at the time of EBL diagnosis, and blood samples had been collected and stored as part of the routine BLV surveillance, and the stored samples were retrospectively used. These samples were previously analyzed for clone dynamics using BLV-capture-seq and BLV-RAIS, as described in our previous publication (6). Detailed information including proviral load, Cvs, numbers of predominant clones in these samples, and the days before EBL diagnosis for blood samples is listed in Table 2. These samples were analyzed using BLV-AFLP. In some cattle, the specific lengths of PCR products were detected at both non-EBL and EBL stages (cattle Nos 7 and 9). The Cvs of these samples were relatively high (0.27 and 0.32, respectively), suggesting that the Cvs of BLV-RAIS reflected the emergence of predominant clones of these sample. These results indicate the sensitivity of BLV-AFLP in detecting clonal dynamics and, except in some cases, the emergence of malignant clones.

**TABLE 2:** Detailed information of cattle analyzed in Fig. 4

	Blood samples			Tumor samples			
	PVL^*a*^	Cv^*b*^	Numbers of dominant clone	tissue	Cv^*b*^	Numbers of dominant clone	The days before diagnosis
1	133,979	0.06	ND^*c*^	Heart	0.6	1	371
2	42,006	0.07	ND	Heart	1	1	374
3	92,480	0.14	ND	Heart	0.8	1	477
4	59,464	0.19	ND	Lymph nodes	0.91	1	387
5	88,909	0.08	ND	Lymph nodes	0.53	2	155
6	143,226	0.06	ND	Heart	0.53	2	169
7	74,114	0.27	ND	Heart	0.61	2	170
8	37,557	0.14	ND	Heart	0.61	2	455
9	68,092	0.32	ND	Heart	0.57	2	462
10	90,296	0.11	ND	Heart	0.54	3	279

^*a*^ Proviral load (copies/10^5^ cells).

^*b*^ Clonality value calculated by BLV-RAIS.

^*c*^ Not detected.

## DISCUSSION

During the progression of EBL, BLV-infected cells clonally expand, and some of which result in tumor onset (4, 6, 7). Therefore, analyzing the clonal dynamics of these cells is useful for diagnosis of EBL progression. As the proviral genome integration site in the host genome serves as a unique marker for each clone of BLV-infected cells, the diversity of host sequences adjacent to the proviral sequences reflects the diversity of BLV-infected cell clones in infected animals. Several approaches, including NGS and Sanger sequencing, have been reported by our groups and others to analyze the diversity of the sequence of BLV integration sites (6, 7, 10, 12). These studies have shown that, in non-EBL cattle, diverse variations in the nucleotide sequence of the host site are detected in the BLV–host chimeric region. On the other hand, due to clonal expansion, few variations in the chimeric region can be observed in EBL cattle, demonstrating that these approaches are feasible for detecting EBL. However, these methods require specialized facilities, laboratory skills, and extensive computational analysis to reconstruct clone structures from deep sequencing or Sanger sequencing data. This complexity makes it difficult to apply these approaches as diagnostic tools in veterinary practice. Measuring proviral load (PVL) is the standard technique for confirming EBL diagnosis, although the sensitivity and specificity of EBL diagnosis were poor in the previous studies (13, 14). Therefore, the development of simple and accurate methods for EBL diagnosis is required.

In this study, we have developed BLV-AFLP, which adopted restriction enzyme-fragmentation and adapter ligation procedure to amplify fragments that include the 3’LTR region of the proviral sequence and host sequence junction. This method allows us to address whether BLV-infected cells proliferate clonally or not, and if so, how many clones are dominantly expanded, without the need for sequencing analysis. By using nested-PCR, followed by agarose gel electrophoresis, analysis of the size of these amplified fragments in non-EBL cattle displayed smeared bands, whereas in EBL cattle, the PCR products are present as a single or at most four distinct bands (Fig. 2–4). The Cvs of these samples were relevant to the PCR band thickness. Furthermore, the number of distinct bands of EBL cattle was mostly identical to that of major predominant clone(s) analyzed by NGS-based BLV-capture-seq (Fig. 2 and 4; the number of clone(s) is shown below the gel images in each figure). These results indicate that BLV-AFLP enables rapid analysis of the clonality of BLV-infected cells.

There were a few cases of inconsistency between BLV-AFLP and BLV-RAIS in both non-EBL and EBL cattle (Fig. 3). For instance, one sample showed a high Cv but presented smeared bands in BLV-AFLP (Fig. 3, non-EBL No. 10, Cv 0.39 with smeared band). The reason why the major clone detected by BLV-RAIS was not amplified by BLV-AFLP remains unclear. One possible explanation for discordance could be the absence of a restriction enzyme site near proviral sequences. BLV-AFLP relies on restriction enzyme fragmentation and PCR amplification. Amplification of the fragment is expected when restriction enzyme sites were placed near proviral 3’LTR sequences, producing a fragment of adequate length for PCR amplification. In this study, we applied restriction enzymes that recognize four nucleotide sequences (*Rsp*RS II and its isoschizomer). These enzymes will cut on average every 256 base pairs in a random sequence. However, the genome contains regions with biased GC content, which do not always conform to this pattern. Another possible explanation is that the fragment containing both the BLV and the host genome might not be amplified if the provirus lacks the primer annealing region of 3’ LTR. However, sequence data of BLV-RAIS analysis revealed that the 3’end of the LTR is clearly detected to the end, ruling this out as a probable cause (data not shown).

In some cases, distinct bands were detected by electrophoresis in non-EBL cattle (Fig. 3, non-EBL Nos 5, 16, 19, and 20). These results are likely due to the sensitive detection of early clone expansion long before tumor onset, which could be increasing in size. Some of these cattle displayed relatively high Cvs of BLV-RAIS, reflecting the slight clonal expansion, although those Cvs were below the cutoff value of EBL diagnosis in our previous report (cutoff value of Cv: 0.4) (6). These results indicated that the BLV-AFLP results needed to be interpreted carefully, along with clinical symptoms and, if necessary, with BLV-RAIS.

The BLV-RAIS method is based on the primer extension reaction of primer set to the BLV3’ LTR region, followed by adapter ligation and nested-PCR. This approach allows for amplification of the chimeric regions of BLV and the host genome irrespective of the nucleotide sequence. However, a notable drawback is that the Cvs can be underestimated, when EBL cattle possess multiple predominant clones or, a single clone with multiple BLV integration because of the sequence diversity of the provirus–host junction. In this study, the cattle diagnosed with EBL by post-mortem inspection exhibited low Cv despite the presence of distinct bands detected by BLV-AFLP (Fig. 3, EBL 2, Cv 0.34 with three distinct bands). Therefore, while the BLV-RAIS method should serve as a universal approach for analyzing clonal proliferation, the BLV-AFLP can be utilized not only for rapid testing to determine the disease status but also for verifying BLV-RAIS results when Cv(s) are near the diagnostic threshold.

In this study, by analyzing cohort samples, we detected the emergence of predominant clones before EBL diagnosis (Fig. 4, cattle No. 7 and No. 9). The same size of distinct bands that were detected in the tumor sample was also detected in the non-EBL stage. In contrast, predominant clones were not detected in the non-EBL stage of other cattle, regardless of the days before EBL diagnosis. These results suggest that the timing of the emergence of the predominant clone in the blood may vary among individual cattle (6, 7). These results also suggest that using BLV-AFLP, we could trace individual clone dynamics in BLV-infected cattle and detect clone numbers in EBL cattle, which is not possible with the BLV-RAIS method.

In this study, we used the NGS library prep kit. A significant advantage of this kit is the one-tube reaction for the end-repair and adapter ligation steps, which substantially reduces reaction time and, consequently, the overall time and personnel cost of the test. However, the cost of a library constructing kit can be the most expensive component in the test process. Therefore, further studies are needed to reduce reagent costs while maintaining generation of unbiased library of input DNA.

This study has several limitations, one of which is that the method becomes unreliable for detecting low PVL samples, due to biased or failed PCR amplification. In this study, the PVLs of non-EBL cattle analyzed in Fig. 2 were higher than 10,000 copies per 10^5^ cells. A previous report has indicated that the samples with PVL less than 500 copies per 10^5^ cells are not applicable for clonal analysis of HTLV-1 (11). Although the samples with low PVL were not analyzed in this study, this limitation also seems to be applied to BLV-AFLP. Another limitation is that the diagnostic discrepancies will occur in EBL cattle that have dominant clones with 3’ end truncated provirus or multiple BLV-infected clones (10, 15). BLV-AFLP cannot distinguish EBL cattle in such cases because the distinct band cannot be detected if the dominant clone(s) are not amplified by PCR.

In summary, we developed BLV-AFLP, which is a simple and rapid method for analyzing the clonal proliferation of BLV-infected cells. The validation using field samples confirmed that BLV-AFLP could effectively identify clonal proliferation in EBL samples. Moreover, this method is capable of tracking clonal dynamics before EBL onset in some cattle, highlighting its sensitivity and potential for early detection. BLV-AFLP is suitable for practical use in the field, improving overall disease management strategies and minimizing economical losses caused by EBL.

## MATERIALS AND METHODS

### Clinical samples

Blood samples were collected from non-EBL cattle on farms by the Shonan Livestock Hygiene Station, Kanagawa Prefectural Government from 2015 to 2023 as a part of national whole herd test surveillance targeting Johne’s disease (Fig. 2A through 4A). These samples were stored at −20℃ until analysis. For the cohort study, tumor samples were collected from EBL cattle, and the corresponding samples from stored blood samples collected from non-EBL cattle were analyzed (Fig. 4). The proviral load was measured by the BLV-CoCoMo-qPCR assay kit (Nippon gene, Tokyo). The whole blood and visually identifiable lymphoma tissues were collected from the carcasses during routine post-mortem inspections by veterinary officers at Meat Inspection Station, Kanagawa Prefectural Government, according to clinical manifestations (Fig. 3B and 4). The detailed sample information of the non-EBL and EBL cattle for Fig. 3 and 4 is provided in Tables 1 and 2, respectively. The study was approved by the ethics committee of Tokyo University of Agriculture. We confirmed that all experiments were performed in accordance with the committee’s guidelines and regulations.

### Genome fragmentation and adaptor ligation

Genomic DNA was extracted from whole blood using the QIAamp DNA Blood Mini Kit (QIAGEN). Genomic DNA was extracted from tumor tissue using a DNeasy Blood and tissue kit (QIAGEN, Germany). Genomic DNA (300 ng) was fragmented using the restriction enzyme *Rsp*RS II (Takara Bio, Japan) at 60°C for 1 hour (Fig. 2) or its isoschizomer *Mse* I (New England Biolabs) at 37°C for 5 minutes (Fig. 3 and 4) End prep and adapter ligation were performed using the NEBNext UltraII DNA Library Prep Kit for Illumina (New England Biolabs, USA) and NEBNext Multiplex Oligos for Illumina (New England Biolabs, USA) according to the manufacturer’s protocol. Briefly, end repair, 5’ phosphorylation, and dA-tailing reactions were performed on the fragmented DNA using NEBNext Ultra II End Prep Enzyme Mix. After an incubation at 20°C, adapter ligation was performed using NEBNext adapter with a hairpin structure and an uracil base. Subsequently, Uracil-Specific Excision Reagent (USER enzyme) is added to generate a single-nucleotide gap at the location of an uracil. Adapter-ligated DNA fragments with Illumina p7 adapter sequence at the 3’ end were cleaned up with column using the QIAquick PCR Purification Kit (QIAGEN) and further digested with restriction enzyme *Fba* I (Takara Bio, Japan) at 37°C for 1 hour (Fig. 2) or its isoschizomer *Bcl* I (New England Biolabs, USA) at 37°C for 5 minutes (Fig. 3 and 4).

### Nested-PCR

The first PCR was performed in a 50-µL reaction mixture containing 5 µL adapter ligated DNA, 5 µL 10X Buffer for KOD-Plus-Neo (Toyobo, Japan), and 0.3 µM forward and reverse primers. The second PCR was performed in a 50-µL reaction mixture containing 1 µL of first PCR products, 5 µL 10X Buffer for KOD-Plus Neo, 0.2 mM each of dNTP, 1U of KOD-Plus-Neo, 1.5 mM of MgSO_4,_ and 0.3 µM forward and reverse primers. The cycling conditions for first round PCR were 94°C for 2 minutes, 30 cycles of 98°C for 10 seconds, and 68°C for 43 seconds, followed by an infinite hold at 4°C. The cycling conditions for second round PCR were 98°C for 30 seconds, 30 cycles of 98°C for 10 seconds, 64°C for 20 seconds, and 72°C for 20 seconds, followed by 72°C for 2 minutes and an infinite hold at 4°C. The forward primer for first PCR was set to recognize common sequences between 10 genotypes of BLV, and the reverse primer was set to the adapter sequence with p7 sequence: 1^st^_For: 5′-CTT GCA CCC RCG TTY GTT TC-3′, 1^st^_Rev: 5’- CAA GCA GAA GAC GGC ATA CGA GAT CGT GAT GTG ACT GGA GTT CAG ACG TGT GCT CTT CCG ATC T-3′. The forward primer was set to the second internal sequence of first PCR products, and the reverse primer was set to the p7 part of 1^st^_Rev primer sequence:2^nd^_For: 5′-CTT ACT TTC TGT TTC TCG CGG C-3′, 2^nd^_Rev:5’- CAA GCA GAA GAC GGC ATA CGA GAT-3′

### Agarose gel electrophoresis

The nested-PCR product was mixed with loading buffer (Takara Bio, Japan) and loaded into wells of a 1% agarose gel. After electrophoresis, the agarose gel was stained with ethidium bromide, and DNA was visualized with UV light.

### BLV-capture-seq

BLV-capture-seq and mapping of viral integration sites were performed as previously described (6, 7). Briefly, genomic DNA was sonicated using a Picoruptor (Diagenode s.a., Liège, Belgium), and then DNA libraries were prepared using a NEBNext Ultra II DNA Library Prep Kit and NEBNext multiplex Oligos for Illumina (New England Biolabs, USA). BLV-specific probes were used for the enrichment of DNA libraries with BLV sequence, and the enriched libraries were PCR-amplified. The enriched DNA libraries were analyzed on the Illumina MiSeq or NextSeq platform. The FASTQ files obtained from the Illumina sequencing were aligned to the Bos taurus reference genome sequence (ARS-UCD1.2/bosTau9) with BLV (GenBank: EF600696) as a separate chromosome or integrated provirus using the BWA-MEM algorithm (16). The chimeric reads containing both the BLV and the host genome were extracted to identify the locations of BLV integration sites in the host genome using the method described previously (7, 17). The number of final virus–host reads in a certain genomic region reflects the initial cell number for each infected clone that enabled us to estimate the clonal abundance for each infected clone (18). BLV whole-genome sequences were obtained from consensus sequence reads mapped to BLV reference sequence and phylogenetically analyzed for genotyping in the previous papers (7, 19).

### Clonality analysis by the BLV-RAIS method

Amplification of the host genome sequence flanking BLV 3’LTR was performed by the BLV-RAIS method as previously described (6). The amplified fragments were then purified using the QIAquick PCR purification kit (QIAGEN) and Sanger sequenced by a commercial DNA sequencing service (FASMAC Co., Ltd.). The Sanger sequence data of the BLV integration sites were analyzed using CLOVA (https://fasmac.co.jp/en/rais) providing 20 nucleotide sequences of the 3’ end of BLV LTR as the transgene sequence (12). Compared with the transgene, CLOVA generates a signal–noise chromatogram for the whole sequence and automatically calculates the clonality value (Cv) taking into consideration the value of the signal peak area (intensity) of the host side and the virus side. The Cv ranges between 0 and 1; 0 indicates the host genome sequence is a contribution of multiple clones with an equal proportion of the load, and 1 indicates the host genome sequence is attributed by a single clone that dominates completely.
